# Relative costing of analytical systems

**DOI:** 10.1155/S1463924680000399

**Published:** 1980

**Authors:** H. G. J. Worth

**Affiliations:** Department of Clinical Chemistry Northwick Park Hospital and Clinical Research Centre Harrow Middlesex HA1 3UJ UK


### Relative costing of analytical systems

H.G.J. Worth
*Department of Clinical Chemistry, Northwick Park Hospital and Clinical Research Centre, Harrow, Middlesex, HA1 3UJ*

### Introduction

Within the last two to three years most hospital departments in the British National Health Service (NHS) have been required to operate within a fixed budget, the preparation of which can be very complex in departments with employees of diverse function and expertise. There is also an increasing demand that the NHS should examine the cost effectiveness of its service; similar demands are being made in other countries. Therefore, a department with a specific function should know the cost of its service, and adjust its budget with strict regard to the quality of cost efficiency of the service.

In 1975 the British Department of Health and Social Security (DHSS) commissioned the preparation of a document outlining a scheme for the "total costing" of a pathology laboratory [1]. Costing exercises in clinical chemistry have since been based on this on this rather complicated scheme or have been concerned solely with the operation of a particular instrument. The latter analysis is often superficial, taking into account only the cost of reagents. If carried out by the manufacturer, the methods of cost analysis can be obscure and difficult to apply to a particular laboratory's workload or to the comparison of the performance of similar instruments marketed by different manufacturers. As capital and running costs of instruments increase it becomes more important to know the relative costs of carrying out analyses by alternative instrumentation before purchasing new equipment.

This study describes a new approach to costing the workload of a hospital laboratory which is less complex than the total costing scheme of Coopers and Lybrand [1]. The scheme presented provides a facility for costing a defined laboratory workload on different instruments, and hence it is referred to as Relative Costing of Analytical Systems (RCAS). It takes into account those costs which are likely to arise in normal circumstances and vary between instruments.

If the workload is made up of a sufficiently broad spectrum of analyses, then not only the costs of single instruments of similar capacity, but also those of combinations of instruments of different capacity can be compared. Such combinations are referred to as systems. It is essential that the workload is defined before RCAS is applied, and for the costing to be meaningful the group of analytes under consideration must constitute a sufficiently large part of the workload. In theory, costing can be carried out on a smaller workload, but in practice this is more difficult as data would have to be corrected to allow for work sharing of instruments, manpower, and in some cases disposables and reagents. Certain assumptions have to be made which must be clearly defined at the outset; they must not invalidate the costing.

### The principles of the cost analysis

The analysis is divided into two parts:
(i) costing of individual instruments
(ii) use of this data to cost different analyitical systems

### Individual instruments

The costing of instruments and their analyses includes all items of expenditure which are not part of the fixed laboratory overheads. These are considered under capital cost, maintenance, manpower, services, control and standardising materials, disposables and reagents.

Costings are calcuated in three categories:—
(a) Costs associated with the instrument regardless of the magnitude or complexity of the workload.
(b) Costs related to batches of analyses.
(c) Costs related to individual specimens.

Each item of expenditure is assessed in the most appropriate category, for example, capital costs are assessed in category (a) and reagent costs in category (c). The appropriate category for other items cannot be specified as it will vary from one instrument to another. When the analysis is complete, two sets of data are available for each instrument, the expenditure related to the instrument ie the sum of the costs in category (a); and expenditure related to the workload ie the sum of the costs in categories (b) and (c).

### Analytical systems

The cost of an analytical system is the sum of the costs of the individual instruments included in the system, ie all the expenditure in category (a) and the expenditure in categories (b) and (c) which correspond to the appropriate parts of the workload. The cost per test is not a fixed sum for an analyte measured by a particular instrument, but is influenced by the way the instrument is used, that is, if the instrument is used to measure this particular analyte only, or in combination or alternation with other analytes.

The overheads excluded from RCAS include personnel indirectly involved with the instrumentation such as senior medical, scientific and technical staff, process workers and secretarial staff, also laboratory overheads such as building costs, heating, lighting, telephone etc.

### Workload

The annual workload of the Clinical Chemistry Department at Northwick Park Hospital has been chosen as a model for

**Table 1. Annual workload model**

| Analyte | Batched | Non batched | Total |
|---|---|---|---|
| Acid phosphatase | 564 | | 564 |
| Albumin | 42,684 | | 42,684 |
| Alkaline phosphatase | 28,346 | | 28,346 |
| Amylase | 2,052 | 156 | 2,208 |
| Aspartate transaminase | 28,452 | | 28,452 |
| Bicarbonate | 8,232 | 2,184 | 10,416 |
| Bilirubin (total) | 25,404 | 240 | 25,644 |
| Bilirubin (conjugated) | 276 | 204 | 480 |
| Calcium | 29,580 | 924 | 30,504 |
| Chloride | 1,392 | | 1,392 |
| Cholesterol | 10,848 | | 10,848 |
| Creatine kinase | 8,736 | | 8,736 |
| Creatinine | 11,628 | 36 | 11,664 |
| Gamma glutamyl transferase | 2,604 | | 2,604 |
| Glucose | 21,732 | 1,404 | 23,136 |
| Iron | 8,064 | | 8,064 |
| Iron binding capacity | 8,064 | | 8,064 |
| Lactate dehydrogenase | 612 | | 612 |
| Phosphate | 28,056 | | 28,056 |
| Potassium | 54,984 | 3,264 | 58,248 |
| Sodium | 54,984 | 3,264 | 58,248 |
| Total protein | 42,540 | | 42,540 |
| Triglyceride | 2,472 | | 2,472 |
| Urate | 11,928 | | 11,928 |
| Urea | 53,004 | 2,772 | 55,776 |
| Multichannel profile | 45,252 | | 45,252 |

the application of RCAS as it is typical for an average sized district general hospital. It was determined on the basis of a detailed study of the analytical data for one month. Work dealt with during the normal working hours is referred to as batched analysis while emergency requests made out of normal working hours are handled separately and referred to as non-batched analyses. For the purpose of RCAS it is assumed that non-batched analyses are carried out as separate, single assays, but samples assayed on Saturday or Sunday mornings are included among the batched analysis workload.

Table 1 contains a list of the analytes considered suitable for inclusion in the study. At least 40 requests were received for each per month and the methods chosen were all capable of being automated. They were measured by the 'kinetic' or 'end point' type of analysis employed in the particular instruments included in the study. Therefore, thyroxine, oestrogen, cortisol and 5'-nucleotidase had to be excluded from the list, but can be subjected to a separate RCAS analysis. Conjugated bilirubin was included because of the measurement of total bilirubin although its rate of analysis was less than 40 per month.

### Reagent costing

The number of methods per analyte has been limited to two for simplicity. Ideally one method should be costed per analyte but this is only possible if the method is applicable to all the instruments under consideration. However, different instruments sometimes require chemically different methods to measure end-point and the rate of reaction of the same analyte. By restricting consideration to two methods, those chosen may not always have been ideal for each instrument but the costing of the reagents is rendered as comparable as is possible for all the instruments.

Reagent costing was based on bulk buying of reagents and kits at prices quoted for the last quarter of 1978, and in quantities appropriate for the workload. The major suppliers were BDH Chemicals Ltd, Poole, England; Clin Tech Ltd, London SE18 5TF and Boehringer Corporation (London) Ltd, Lewes, England. The proportional quantities of each reagent required in a particular routine method were calculated and the cost per litre of the final reagent mixture determined.

It has been assumed that the identity and relative quantities of the constituents of each final reagent mixture are invariant with respect to different instruments. This assumption is not completely valid, but when the cases with deviations were investigated, the differences in the individual costs of the constituents did not significantly change the cost of the final reaction mixture. The final reagent cost for each method considered is given in Table 2. Some analyses require a blank determination to be carried out and where this involves different reagents the appropriate cost has been calculated separately. This approach to costing does not apply to instruments for which use of the manufacturer's reagents and kits is obligatory eg the Du Pont ACA.

### Manpower

In RCAS only the manpower used in operating the analytical instruments is taken into account. This includes the time that would normally be spent by the operator in day to day maintenance. It is assumed that operating the type of instruments included in this study does not influence intra laboratory requirements in expertise and times of employment of other personnel who do or do not contribute to the processing of specimens.

Manpower costing has been determined in two ways according to the type of instrumentation. Annual costing is used for non-selective multichannel instruments where, assuming the personnel are employed full time on such analyses, the manpower is unaffected by the number of analytes determined. With the smaller instruments whose usage and hence manpower utilisation is affected by the number of analytes measured, hourly costing is applied. The unit time cost has been calculated on the mean salary of the Medical Laboratory Scientific Officer (MLSO) for the last quarter of 1978 plus 20% to cover national insurance and superannuation etc. Calculation of the hourly rate is based on a 37 hour, 5 day week and adjusted for holidays and a 13% deduction for non productive time, ie time not spent on the actual processes of measurement. The following data are derived:–

| | |
|---|---|
| Mean annual MLSO salary (for last quarter of 1978) | £3,970.50 |
| 20% (insurance, superannuation) | £794.10 |
| Total annual manpower cost/person | £4,764.60 |
| Calculation of hourly rate:– | |
| Number of working days (52 x 5) | 260 days/annum |
| Annual leave | 20 days/annum |
| Bank holidays | 8 days/annum |
| Statutory holidays | 2 days/annum |
| Total days lost | 30 days/annum |
| True number of working days | 230 days/annum |
| Number of working hours $230 \times \frac{37}{5}$ | 1,702 hours/annum |
| 13% non productive time | 221 hours/annum |
| Net number of working hours | 1,481 hours/annum |
| Hourly rate $\frac{4764.6}{1481}$ | £3.22/h |

In the case of the non-batched analysis, manpower is covered by a fixed "on-call" payment which is irrespective of the number of analyses carried out and the type of instrument used; it is therefore excluded from this costing. The

**Table 2. Final reagent cost**

| Analyte | Method | Cost (pence/1) |
|---|---|---|
| Acid phosphatase | I Nitrophenyl phosphate | 372.7 |
| Albumin | I Bromocresol green | 38.0 |
| Alkaline phosphatase | I King and Kind | 24.2 |
| | Blank | 21.1 |
| | II Nitrophenyl phosphate | 4770.0 |
| Amylase | I Starch/iodine | 8.2 |
| Aspartate transaminase | I NAD/NADH | 4036.0 |
| | Blank | 4036.0 |
| Bicarbonate | I Cresol red | 2.0 |
| | II Carboxylase | 2491.0 |
| | Blank | 40.6 |
| Bilirubin | I Diazo Michaelsson | 59.9 |
| Calcium | I Cresolphthalein | 50.7 |
| Chloride | I Thiocyanate | 11.7 |
| Cholesterol | I Cholesterol oxidase | 6632.0 |
| | II Leibermann-Burchard | 251.1 |
| Creatine kinase | I NAD/NADH | 23810.0 |
| | II Diacetyl/orcinol | 6414.6 |
| Creatinine | I Jaffe | 17.8 |
| | Blank | 0.8 |
| γ-Glutamyl transferase | I NAD/NADH | 6633.0 |
| | Blank | 6633.0 |
| Glucose | I Glucose oxidase | 108.5 |
| Iron and I.B.C. | I Ferrozine | 59.4 |
| Lactate dehydrogenase | I NAD/NADH | 3800.0 |
| | Blank | 24.7 |
| Phosphate | I Stannous chloride | 54.9 |
| Sodium and potassium | I Flame photometry | 1.2 |
| | II Ion selective electrode | 479.0 |
| Protein | I Biuret | 25.2 |
| | Blank | 20.6 |
| Triglyceride | I NAD/NADH | 11923.0 |
| | Blank | 9200.0 |
| Urate | I Uricase | 29.0 |
| | Blank | 135.5 |
| | II Phosphotungstate | 386.6 |
| Urea | I Berthelot | 103.4 |
| | II Diacetyl monoxime | 51.0 |

manpower costs for Saturday and Sunday mornings are also covered by "on-call" payments. However, the magnitude of the workload at these times is such that it was costed as batched analysis.

### Other costs

The costs other than those of reagents and manpower will be considered here under the appropriate sub-headings.

### Capital and depreciation

The capital cost of an instrument needs to be included and should be spread uniformly through its expected working life. The DHSS recommendation of seven years average life [1] has been used and depreciation has therefore to be calculated as one seventh of the capital cost per annum. To this has to be added the cost of the maintenance contract, which usually stipulates that the instrument be maintained under warranty, free of charge for the first year. Therefore, the total annual depreciation has been calculated as one seventh of the capital cost plus six sevenths of the annual maintenance contract. The latter is usually quoted as a percentage of the capital cost.

### Data processing

The data handling capability of instruments varies greatly and is reflected in their capital cost and manpower requirements for data transfer. This has been allowed for by setting a minimum data processing requirement for each instrument; this is the ability to interface with a computer capable of handling the laboratory's workload. When this requirement is not met by the instrument an addition is made to the capital cost to cover suitable interfacing equipment.

### Services

The services included are electricity, water and gas. Electricity and water are calculated as a product of the estimated mean consumption of the instrument and the local cost per unit, which gives only an approximate cost as the mean consumption cannot be calculated accurately. Water cost is negligible and is ignored in almost all cases except multichannel instruments fitted with a laundry system. Where the water consumption is high the cost of deionising resin is significant and has been included. Gas requirement is solely that of propane for flame photometry. Its cost has been based on purchasing large cylinders appropriate to the size of the workload.

### Commercial sera

Commercial sera used for calibration and control of analyses vary from one laboratory to another and therefore it is difficult to make a generalised statement concerning their cost. The cost calculated here was based on the following assumptions: –

(i) All calibration is carried out using assayed commercial sera.
(ii) Unassayed commercial sera are used for all quality control procedures.
(iii) Quality control specimens are included at the frequency of 1 in 20 for batched analyses and at a frequency of 1 to 1 in non-batched analyses, unless a more appropriate frequency is dictated by the instrument.
(iv) The volume of serum used is approximately 10% in excess of the volume required for the assay.

Costing is based on the mean price of preparations purchased in quantities appropriate to the workload. The case where a special serum was required for a particular instrument is dealt with in the section on instrumentation.

### Disposables

These include blood tubes for the transportation of specimens, vials for introducing the specimen into the instrument, and any accessories associated with the analyser which need

**Table 3. Instrument associated costs of Vickers M–300**

| | | | Annual cost (pounds) |
|---|---|---|---|
| Capital | Capital cost | £125,000 | |
| | Maintenance 8% after 1 yr | | |
| | Capital depreciation | | |
| | $(\frac{125000}{7} + 125000 \times \frac{8}{100} \times \frac{6}{7})$ | | 26,428.57 |
| Manpower | 3 full time personnel at £4,764.60/person | | 14,293.80 |
| Analysis data | Number of specimens/annum | 45,252 | |
| | Number of standards and controls | 70/d | |
| | Total number of assays/annum | | |
| | (45,252 + 70 x 260) | 63,452 | |
| | ∴ Total analysis time/annum | | |
| | $(\frac{63452}{300})$ | 211.5h/annum | |
| | Run up time | 2 h/d | |
| | Run down time | 0.5 h/d | |
| | Total running time/annum | | |
| | (211.5 + (2+0.5) x 260) | 861.5 h/annum | |
| Services | Mean electrical consumption | 15 KVA/h | |
| | Annual cost (consumption x rate x running time) | | |
| | $(15 \times \frac{1.865}{100} \times 861.5)$ | | 241.00 |
| | Water consumption | 601/h | |
| | Annual cost (consumption x rate x running time) | | |
| | $(60 \times \frac{0.0117}{100} \times 861.5)$ | | 6.05 |
| | Annual cost deionisation resin | | 624.00 |
| | Annual gas consumption | 6 units | |
| | Annual cost (6 x 5.50) | | 33.00 |
| Commercial sera | Unassayed serum consumption | 90 m/d | |
| | Annual cost (unit cost x consumption) | | |
| | $(\frac{10.4}{100} \times 90 \times 260)$ | | 2,433.60 |
| | Assayed serum consumption | 12.5 ml/d | |
| | Annual cost (unit cost x consumption) | | |
| | $(\frac{15.0}{100} \times 12.5 \times 260)$ | | 487.50 |
| Disposables | Mean vial cost | 9p ea | |
| | Annual vial cost (unit cost x total assays) | | |
| | $(\frac{9}{100} \times 63{,}452)$ | | 5,710.68 |
| | Blood tubes cost (unit cost x number used) | | |
| | $(\frac{2.64}{100} \times 45{,}252 \times \frac{40}{100})$ | | 477.86 |
| | | Total | 50,736.06 |
| Additional analyses | Standards and controls | 70/d | |
| | Annual analyses (70 x 260) | 18,200 | |
| | Reagent priming time | 24 min/d | |
| | Annual priming time | | |
| | $(\frac{24}{60} \times 260)$ | 104 h | |
| | Equivalent number of analyses for purposes of reagent costing | | |
| | (104 x 300) | 31,200 | |
| | Total additional analyses | | |
| | (18,200 + 31,200) | 49,400 | |
| | Effective annual workload (workload + additional analyses) | | |
| | (45,252 + 49,400) | 94,642 VI | |

replacing at regular intervals, for example pump tubes and dialyser membranes in continuous flow equipment. Vials are usually peculiar to the instrument and are therefore costed individually. In determining the number required, allowances must be made for the controls and the standards, as well as for the specimens. Blood tube cost is based on that of plain glass 10 ml tubes purchased at a bulk rate appropriate to the workload, the consumption being based on one tube for every four tests, reflecting a test/request ratio of 4:1 which the DHSS suggest is the national average [2]. This figure is not used for the multichannel non-selective instruments where it is assumed that one tube is used for each profile. No allowance is made for special tubes required for certain tests, for example glucose.

The cost of disposables such as chart paper, request and result forms are assumed to be invariable within a laboratory irrespective of the equipment, and therefore have been omitted.

### Instrumentation

Ten different types of instrument have been selected for cost analysis. As stated previously, reagents have been costed per unit volume of the final mixture and the reagent cost for an instrument is based on the volume it consumes. All other expenses are calculated to assess the total running cost per annum of the individual instrument. A common costing pattern is used for all instruments, although its application varies with the differences in the individual modes of operation. For example an item may have to be classified as an overhead with one but not necessarily with another. Consequently a series of formulae are derived for each instrument, two examples of which may be seen in Tables 3 and 5. From these data a total overhead cost is determined and a total assay cost per analyte per annum is calculated. The latter is determined for batched analyses and non-batched ones where applicable.

In the case of some of the newer instruments data were not available at the time of compilation for all of the potential analyses, and these analyses have therefore been omitted.

The costs of the materials and services are based on information which was correct at December 1978 and which are common to all instruments, they are shown below.

| | |
|---|---|
| Electricity | 1,8651p/KVA |
| Water | 0.117p/l |
| Gas | £5.50/unit |
| Unassayed commercial sera | 10.4p/ml |
| Assayed commercial sera | 15.0p/ml |
| Blood tubes | 2.64p/ea |
| Blood tube cost per test on the basis of 4 tests per tube (where applicable) | 0.66p/test |

The costs of other materials which are required specifically for certain instruments are accounted for individually.

Two examples of costing have been given in Tables 3 to 6 which demonstrate two extremes of approach. The Vickers M300 multichannel analyser is a profiling instrument and most of its costing is related to the instrument rather than the workload, whereas the converse is true for the Union Carbide Centrifichem 400.

**Table 4. Analyte associated costs of Vickers M-300**

| | Method | Sample volume (μl) | Reagent volume (ml) | Reagent cost/1 (pence) | Cost/test (pence) | Total cost (pounds) |
|---|---|---|---|---|---|---|
| Column | I | II | III | IV | V | VI |
| Derivation | see Table 2 | | | see Table 2 | $\frac{III}{1000} \times IV$ | $\frac{V}{100} \times 94652$ |
| Acid phosphatase | | | | | | |
| Albumin | I | 7 | 3.50 | 38.0 | 0.133 | 125.89 |
| Alkaline phosphatase | I | 58 | 3.50 | 24.2 | 0.0847 | 80.17 |
| Blank | I | 58 | 3.50 | 21.1 | 0.0738 | 69.86 |
| Amylase | | | | | | |
| Aspartate transaminase | I | 58 | 2.50 | 4036.0 | 10.090 | 9550.39 |
| Blank | I | 58 | 2.50 | 4036.0 | 10.090 | 9550.39 |
| Bicarbonate | II | 3 | 2.50 | 2491.0 | 6.2275 | 5894.44 |
| Blank | II | 3 | 2.50 | 40.6 | 0.1015 | 96.05 |
| Bilirubin (total) | I | 133 | 3.50 | 59.9 | 0.2097 | 240.10 |
| Bilirubin (conjugated) | I | 133 | 3.50 | 59.9 | 0.2097 | 240.10 |
| Blank | I | 133 | 3.50 | 59.9 | 0.2097 | 240.10 |
| Calcium | I | 30 | 3.50 | 50.7 | 0.1775 | 169.30 |
| Chloride | I | 3 | 3.50 | 11.7 | 0.0410 | 38.80 |
| Cholesterol | II | 58 | 2.50 | 251.1 | 0.6278 | 594.22 |
| Creatine kinase | | | | | | |
| Creatinine | I | 133 | 3.50 | 17.8 | 0.0623 | 58.98 |
| Blank | I | 133 | 3.50 | 0.8 | 0.0028 | 2.65 |
| Gamma glutamyl transferase | I | 58 | 2.50 | 66330.0 | 16.5835 | 15696.66 |
| Blank | I | 58 | 2.50 | 66330.0 | 16.5825 | 15695.66 |
| Glucose | I | 20 | 4.00 | 108.5 | 0.4340 | 410.78 |
| Iron | | | | | | |
| Iron binding capacity | | | | | | |
| Lactate dehydrogenase | I | 25 | 3.50 | 3800.0 | 13.3000 | 12588.72 |
| Blank | I | 25 | 3.50 | 24.7 | 0.0865 | 81.87 |
| Phosphate | I | 17 | 3.50 | 54.9 | 0.1922 | 181.91 |
| Potassium ) Sodium ) | I | 15 | 3.60 | 1.2 | 0.0043 | 4.06 |
| Total Protein | I | 33 | 3.50 | 25.2 | 0.0882 | 83.48 |
| Blank | | | | | | |
| Triglyceride | | | | | | |
| Blank | | | | | | |
| Urate | I | 20 | 3.40 | 29.0 | 0.0986 | 93.33 |
| Blank | I | 20 | 3.40 | 135.5 | 0.4607 | 436.07 |
| Urea | I | 4 | 3.50 | 103.4 | 0.3619 | 342.55 |

The results of cost analyses carried out on ten different instruments are shown in Table 7.

### Systems costing

Different instruments and/or multiples of the same instrument used in combination to process a defined workload are referred to as systems. The costing of a system is carried out by summing the relevant individual instrument costs as in Table 7. A number of examples of systems costings are given below.

### Workload/cost comparison

The analytes included in the batched workload shown in Table 1 have been rearranged according to the magnitude of the workload commencing with the largest. Each is then costed for each applicable instrument and these costs which are cumulative, are summed. Figure 1 shows the results of this costing procedure diagramatically. The point at which the plot meets the abscissa represents the instrument associated costs. Thus the expensive multichannel instruments have poor cost effectiveness at low workloads, although in this case it is low because not all the channels are in use. With the smaller instruments a point is reached where no more work can be handled during the normal working day and so an additional instrument is required with an additional increase in capital expenditure. Therefore, it is necessary to determine the analysis time and the total time available during the normal working day. The latter has been calculated as follows:–

| | |
|---|---|
| Number of working days (52 x 5) | 260 days/annum |
| Bank holidays | 8 days/annum |
| Statutory holidays | 2 days/annum |
| Total days lost | 10 days/annum |

**Table 5. Instrument associated costs of Union Carbide Centrifichem 400**

| | | | Annual Cost (pounds) |
|---|---|---|---|
| Capital | Capital cost | £37,500 | |
| | Maintenance 8% after 1 yr<br>Capital depreciation<br>$(\frac{37.500}{7} \times 37{,}500 \times \frac{8}{100} \times \frac{6}{7})$ | | 7,928.57 |
| Manpower | 1 Full time operator at | £3.22/h | |
| Analysis data | Analysis time<br>Total number analyses/rotor<br>Priming reagent volume<br>Plate changeover time<br>Plate sampling time | variable<br>29<br>None<br>10 min<br>4 min | |
| Services | Electrical consumption<br>Cost (consumption x rate)<br>(4.8 x 1.865)<br>Laundry water + deionising resin<br>Methanol | 4.8KVA/h<br><br>8.952p/h<br>1.3p/l<br>55p/l | |
| Commercial sera | Unassayed serum consumption<br>Cost (unit cost x consumption)<br>$(10.4 \times \frac{100}{1000})$<br>Assayed serum consumption<br>Cost (unit cost x consumption)<br>$(15.0 \times \frac{100}{1000})$ | 100µl/test<br><br>1.04p/test<br>100 µl/test<br><br>1.5p/test | |
| Disposables | Analyser cups<br>Blood tubes (4 tests/tube)<br>$\frac{(2.64)}{4}$ | 0.55p/ea<br><br>0.66p/ea | |
| | | Total | 7,928.57 |
| Batched analysis | Reagent usage/rotor (2 unassayed sera + 2 assayed sera + 25 specimens)<br>(Reagent volume + 16% reagent volume)<br>Corrected reagent cost/test<br>cost/test + 16% cost/test | | VI |
| | Total analysis time/rotor (2 unassayed sera analysis time + 2 assayed sera analysis time + 1 blank analysis time + 25 specimen analysis time + 10 min changeover + 4 min sampling time)<br>Corrected analysis time/specimen<br>Analysis time + 20% analysis time + $\frac{14}{25}$ min | | X |
| | Total manpower cost (unit cost x total running time)<br>(3.22 x total running time)<br>Total electrical cost (unit cost x total running time)<br>$(\frac{8.952}{100}$ x total running time) | | |
| Batched analysis (cont'd.) | Manpower/electrical cost $(3.22 + \frac{8.952}{100})$ total running time<br>3.31 x total running | | XI |
| | Rotor costs (25 specimens)<br>(2 assayed sera + 2 unassayed sera + 29 analyser cups + 25 (¼) blood tubes)<br>(1.5 x 2 + 1.04 x 2 + 0.55 x 29 + 0.66 x 25) 37.53p/rotor<br>Rotor cost/specimen<br>$\frac{37.53}{25}$ 1.50121/specimen | | XII |
| | Laundry cost/rotor<br>Water + resin (100 ml) 0.13p/rotor<br>Methanol (40 ml) 2.2p/rotor<br>Water + resin + methanol 2.33p/rotor | | XXII |
| | Cost/specimen<br>$\frac{2.33}{25}$ 0.0932p/specimen | | XXII |
| Non batched assays | Reagent usage/specimen (2 assayed sera + 1 unassayed serum + specimen)<br>Reagent volume + 300% reagent volume)<br>Corrected reagent cost/test<br>cost/test x 4 | | XV |
| | Total analysis time/specimen<br>(2 assayed sera analysis time + 1 unassayed sera analysis time + 1 blank analysis time + 1 specimen analysis time + 10 min changeover)<br>(sampling time is excluded as rotor is only partially filled)<br>Corrected analysis time/specimen<br>analysis time x 5 + 10 min | | XIX |
| | Manpower cost not applicable<br>Total electrical cost (unit cost x total running time)<br>$\frac{8.952}{100}$ x total running time | | XX |
| | Rotor cost/specimen<br>(2 assayed sera + 1 unassayed serum + 4 analyser cups + 1 (¼) blood tube)<br>(1.5 x 2 + 1.04 + 0.55 x 4 + 0.66)<br>6.9p/specimen<br>Laundry cost/rotor<br>For non batched analysis the cost/rotor and cost/specimen are the same, i.e. 0.0932p/specimen | | |

| | |
|---|---|
| True number of working days | 250 days/annum |
| Number of working hours (250 x $\frac{37}{5}$) | 1,850 h/annum |
| 13% (non productive time) | 240.5 h/annum |
| Maximum analysis time | 1,609.5 h/annum |

In Figure 1 the points of discontinuity of some graphs indicate the stage where an additional instrument is required. The points of inflection occur where the analyte associated costs are higher than the average for that instrument.

**Multichannel instruments**

The total cost of running each of the four multichannel instruments at full capacity is compared. Except for the Hycel-M none of these is capable of analysing the complete workload model and therefore a suitable selection of analytes is made as shown in parenthesis below. For the SMAII system, costing is based on a 16 channel instrument including a flame unit. Costs are as follows :-

Vickers M-300 £78,405.90 (Albumin, alkaline phosphatase, aspartate transaminase, bicarbonate, total bilirubin, calcium, chloride, creatinine, glucose, phosphate, potassium, sodium, total protein, urate, urea)

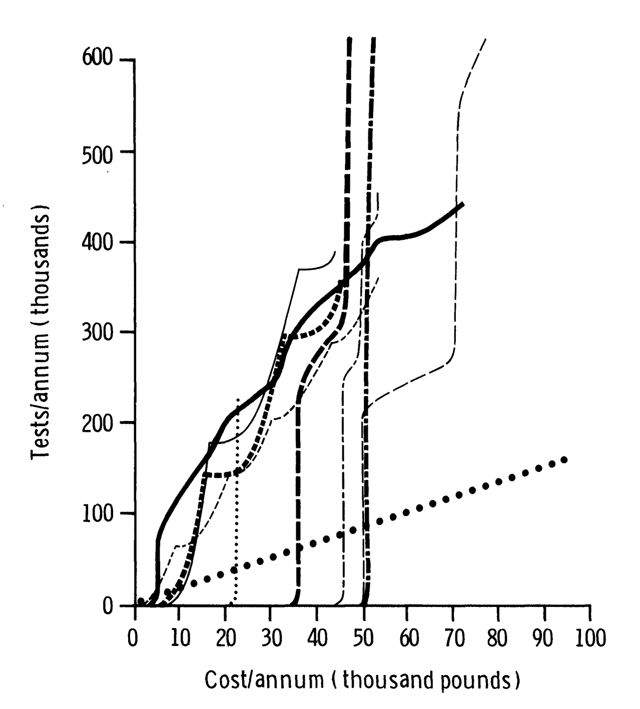

*Figure 1. Annual test/cost relationship.*

*Key to Figure 1.*

- –·–·– Hycel M

- ············ Technicon SMAII (6 channels)

- ▬ ▬ ▬ ▬ Technicon SMAII (16 channels)

- ▬▬▬▬ Technicon AAII

- ------- Coulter Kem-O-Mat

- ——— Abbot VP

- ▪▪▪▪▪▪▪▪ Centrifichem 400

- ●●●●●● DuPont ACA

- ——— Vickers M-300

- —·—·— Technicon SMAC

**Table 6 Analyte associated costs of Union Carbide Centrifichem 400**

| | Method | Sample volume (µl) | Reagent volume (ml) | Reagent cost/1 (pence) | Cost/test (pence) | Corrected reagent cost/test (pence) | Annual workload | Reagent annual cost (pounds) | Analyses (min/spec) | Total running time (h/annum) |
|---|---|---|---|---|---|---|---|---|---|---|
| Column | I | II | III | IV | V | VI | VII | VIII | IX | X |
| Derivation | see Table 2 | | | see Table 2 | $\frac{III}{1000}$ x IV | V + 16% V | see Table 1 | $\frac{VI}{100}$ x VII | | (IX+20%IX 14) VII / 25) 60 |
| Acid phosphatase | | | | | | | | | | |
| Albumin | I | 3 | 0.407 | 38.0 | 0.0155 | 0.0180 | 42684 | 7.68 | 0.133 | 511.9 |
| Alkaline phosphatase | II | 5 | 0.305 | 4770.0 | 1.4549 | 1.6877 | 28236 | 476.54 | 0.033 | 282.2 |
| Blank | | | | | | | | | | |
| Amylase | | | | | | | | | | |
| Aspartate transaminase | I | 40 | 0.305 | 4036.0 | 1.2310 | 1.4280 | 28452 | 406.29 | 0.033 | 284.3 |
| Blank | | | | | | | | | | |
| Bicarbonate | | | | | | | | | | |
| Blank | | | | | | | | | | |
| Bilirubin (total) | I | 25 | 0.305 | 59.9 | 0.0183 | 0.0212 | 25404 | 5.39 | 0.133 | 304.7 |
| Bilirubin (conjugated) | I | 40 | 0.305 | 59.9 | 0.0183 | 0.0212 | 276 | 0.06 | 0.033 | 2.8 |
| Blank | I | – | 0.305 | 59.9 | 0.0183 | 0.0212 | 25404 | 5.39 | 0.033 | 253.9 |
| Calcium | I | 5 | 0.405 | 50.7 | 0.02053 | 0.0238 | 29580 | 9.04 | 0.033 | 295.6 |
| Chloride | | | | | | | | | | |
| Cholesterol | I | 5 | 0.375 | 6632.0 | 2.487 | 2.8849 | 10848 | 312.96 | 0.500 | 209.7 |
| Creatine kinase | I | 15 | 0.385 | 2380.0 | 9.1669 | 10.6336 | 8736 | 928.95 | 0.033 | 116.2 |
| Creatinine | I | 40 | 0.405 | 17.8 | 0.0072 | 0.0084 | 11628 | 0.98 | 0.033 | 116.2 |
| Blank | | | | | | | | | | |
| $\gamma$glutamyl transferase | I | 25 | 0.305 | 6633.0 | 2.0231 | 2.3468 | 2604 | 61.11 | 0.033 | 26.0 |
| Blank | | | | | | | | | | |
| Glucose | I | 5 | 0.305 | 108.5 | 0.0331 | 0.0384 | 21732 | 8.35 | 0.200 | 289.8 |
| Iron | I | 100 | 0.250 | 59.4 | 0.0149 | 0.0173 | 8064 | 1.40 | 0.333 | 129.0 |
| Iron binding capacity | I | 100 | 0.250 | 59.4 | 0.0149 | 0.0173 | 8064 | 1.40 | 0.333 | 129.0 |
| Lactate dehydrogenase | I | 10 | 0.300 | 3800.0 | 1.1400 | 1.3224 | 612 | 8.09 | 0.033 | 6.1 |
| Blank | | | | | | | | | | |
| Phosphate | | | | | | | | | | |
| Potassium | | | | | | | | | | |
| Sodium | | | | | | | | | | |
| Total protein | I | 10 | 0.405 | 25.2 | 0.0102 | 0.0118 | 42540 | 5.02 | 0.033 | 425.1 |
| Blank | | | | | | | | | | |
| Triglyceride | I | 5 | 0.375 | 1192.0 | 0.4470 | 0.5183 | 2472 | 12.82 | 0.0333 | 39.5 |
| Blank | | | | | | | | | | |
| Urate | I | 10 | 0.300 | 29.0 | 0.0087 | 0.0101 | 11928 | 1.21 | 0.167 | 151.2 |
| Blank | I | 10 | 0.300 | 135.5 | 0.0405 | 0.0470 | 11928 | 5.61 | 0.167 | 151.2 |
| Urea | I | 5 | 0.375 | 103.4 | 0.0388 | 0.450 | 53004 | 23.85 | 0.008 | 503.2 |

Columns VI to XIV refer only to batched analysis Columns XV to XIX refer only to non-batched analysis

| | | |
|---|---|---|
| Technicon SMAC | £62,212.60 | Albumin, alkaline phosphatase, aspartate transaminase, bicarbonate, total and conjugated bilirubin, calcium, chloride, cholesterol, creatine, kinase, creatinine, glucose, lactate dehydrogenase, phosphate, potassium, sodium, total protein, triglyceride, urate, urea) |
| Hycel-M | £53,700.14 | |
| Technicon SMAII | £49,178.57 | (same as Vickers M-300) |

These figures are not directly comparable for two reasons. The number of analytes determined is different; 15 with the M-300, 20 with the SMAC, 25 with the Hycel-M and 15 with the SMAII. The Hycel-M assays the complete workload model whereas the other three non-selective instruments cover only the profile workload defined by their capacity. The difference, therefore, between the performance of Hycel-M and the others is the total workload minus the respective profile workloads which is approximately 10,000 assays for sodium, potassium, bicarbonate and urea, plus the non-batched workload.

**Systems using smaller instruments**

Examples of systems capable of carrying out the batched and non-batched workload are shown below using different combinations of Kem-O-Mat, Abbott VP and Centrifichem. The cost of an AAII flame unit is included as none of these instruments is capable of sodium and potassium analysis.

AAII Flame Unit + 5 Kem-O-Mats

| | |
|---|---|
| Flame Unit | £5,154.18 |
| Kem-O-Mat | £57,320.40 |
| Total/annum | £62,474.58 |

The analytes excluded are acid phosphatase, amylase, bicarbonate, $\gamma$–glutamyl transferase, iron and IBC.

AA11 Flame Unit + 3 VPs

| | |
|---|---|
| Flame Unit | £5,154.18 |
| VP Instruments | £43,878.70 |
| Total/annum | £49,032.88 |

Acid phosphatase is the only analyte exluded.

AAII Flame Unit + 3 Centrifichems

| | |
|---|---|
| Flame Unit | £5,154.18 |
| Centrifichems | £46,932.03 |
| Total/annum | £52,086.21 |

The analytes excluded are acid phosphatase, amylase, bicarbonate, chloride and phosphate.

AAII Flame Unit + Kem-O-Mat + 2 VPs

| | |
|---|---|
| Flame Unit | £5,154.18 |
| Kem-O-Mat | £9,825.22(Creatine, urea) |
| VPs | £33.408.70(rest of the analyses) |
| Total/annum | £48.388.10 |

Acid phosphatase is the only analyte excluded.

**Table 6.continued**

| Electrical manpower cost/annum (pounds) | Specimen cost/ annum (pounds) | Laundry cost/ annum (pounds) | Total cost/ annum (pounds) | Corrected reagent cost/test (pence) | Annual workload | Annual cost reagent (pounds) | Analysis time (min/ spec) | Total running time (h/annum) | Electrical cost/ annum (pounds) | Specimen cost/ annum (pounds) | Laundry cost/ annum (pounds) | Total cost/ annum (pounds) |
|---|---|---|---|---|---|---|---|---|---|---|---|---|
| XI | XII | XIII | XIV | XV | XVI | XVII | XVIII | XIX | XX | XXI | XXII | XXIII |
| 3.31 x X | $\frac{1.5012}{100}$VII | $\frac{0.0932}{100}$VII | VIII+XI+XII +XIII | 4V | see Table 1 | $\frac{XV}{100}$x XVI | as IX | (5xVIII+10) $\frac{XVI}{60}$ | $\frac{8.952}{100}$x XIX | $\frac{6.9}{100}$XVI | $\frac{2.33}{100}$XVI | XVII+XX+XXI+XXI |
| 1696.39 | 640.77 | 39.78 | 2382.62 | | | | | | | | | |
| 934.08 | 423.88 | 26.31 | 1860.81 | | | | | | | | | |
| 941.03 | 427.12 | 26.55 | 1800.99 | | | | | | | | | |
| 1008.58 | 381.36 | 23.71 | 1419.04 | 0.0732 | 240 | 0.18 | 0.133 | 42.7 | 3.82 | 16.56 | 5.92 | 26.48 |
| 9.27 | 4.14 | 0.23 | 13.70 | 0.0732 | 204 | 0.15 | 0.033 | 34.6 | 3.10 | 14.08 | 4.75 | 22.08 |
| 840.41 | 381.36 | 23.71 | 1250.87 | 0.0732 | 240 | 0.18 | 0.033 | 40.7 | 3.64 | 16.56 | 5.92 | 26.30 |
| 978.44 | 444.05 | 27.57 | 1457.10 | 0.0821 | 924 | 0.76 | 0.033 | 156.5 | 14.01 | 63.76 | 21.53 | 100.06 |
| 694.11 | 162.85 | 10.08 | 1180.00 | | | | | | | | | |
| 657.37 | 131.14 | 8.11 | 1725.57 | | | | | | | | | |
| 384.62 | 174.56 | 10.88 | 571.04 | 0.0288 | 36 | 0.01 | 0.033 | 6.1 | 0.55 | 2.48 | 0.84 | 3.88 |
| 86.06 | 39.09 | 2.44 | 188.70 | | | | | | | | | |
| 959.24 | 326.24 | 20.24 | 1314.07 | 0.1324 | 1404 | 1.86 | 0.200 | 237.9 | 21.30 | 96.88 | 32.71 | 152.75 |
| 427.00 | 121.06 | 7.48 | 556.94 | | | | | | | | | |
| 427.00 | 121.06 | 7.48 | 556.94 | | | | | | | | | |
| 20.26 | 9.19 | 0.55 | 38.09 | | | | | | | | | |
| 1407.08 | 638.61 | 39.70 | 2090.41 | | | | | | | | | |
| 130.75 | 37.11 | 2.29 | 182.97 | | | | | | | | | |
| 500.47 | 179.06 | 11.11 | 691.85 | | | | | | | | | |
| 500.47 | 179.06 | 11.11 | 692.25 | | | | | | | | | |
| 1665.59 | 795.70 | 49.47 | 2534.61 | 0.1552 | 2773 | 4.30 | 0.008 | 496.6 | 42.04 | 191.27 | 64.59 | 302.20 |

AAII Flame Unit + 2 Kem-O-Mats + 2 Centrifichems

| | |
|---|---|
| Flame Unit | 5,154.18 |
| Kem-O-Mats | £18,876.84(Calcium, chloride, creatinine, phosphate, urea) |
| Centrifichems | £34,034.57(rest of the analyses) |
| Total/annum | £58,065.59 |

The analytes excluded are acid phosphatase, amylase and bicarbonate.

### Conclusions

A method is proposed for assessing the cost of the annual workload and the capital depreciation of an instrument or instruments. The protocol has been standardised as much as possible so that it is easily applicable to all instruments currently used or to be used in clinical chemistry laboratories, individually or comparatively. This is one of the main advantages of RCAS over cost analyses carried out elsewhere as part of instrument evaluations on some instruments considered in the present study [3-5].

In the application of RCAS certain assumptions are made, particularly with respect to overheads which have been considered to be independent of the operation of the instruments. If at any time for any reason, one of these assumptions is considered invalid, suitable adjustments can be made to the cost analysis. A capital depreciation allowance is made for data processing equipment as an accessory to some instruments in order to level their performance with that of more advanced models which can be interfaced with a computer. It was not intended here, however, to include an analysis of computer costing. This, and the overheads referred to above are assumed to be independent of instrumentation and could be the subjects of separate RCAS programmes. A combination of all three would give a total costing similar to that of Coopers and Lybrand [1].

Ten types of instrument have been costed covering the range which is, or soon will be, available in the UK to the clinical chemist for the analysis of end point and/or rate of reaction. Combinations of these instruments have been costed for the execution of a defined workload with the general conclusion that the more recently introduced instrumentation is more economic, and that if the correct combination is used it makes little difference whether the system is based on a large multichannel analyser or a series of smaller discrete instruments. The costing of the newer instruments is based more on the theory than the practice of operation and therefore it is possible that the data used give a falsely low costing.

One of the most important aspects of instrumentation not considered by RCAS or other costing procedures is the quality of analysis. This cannot be evaluated mathematically but it can be assessed comparatively for different instruments and therefore provides a basis for justification of the cost of a particular chemical method or instrument.

The equipment and systems covered in this paper represent only a small part of the total number available but the study was laborious and time consuming. It is suggested that RCAS would operate most effectively through a computer program. It is also recommended that a costing file containing all the information concerning the cost of capital, reagents, disposable items, service and manpower should be kept and updated periodically to allow for price increases. A separate file could contain instrumentation details with the new information entered as it becomes available. A relative cost analysis could then be carried out at any time for a given workload using any specified combination of instruments. The compilation of such a program would make it easier

**Table 7. Instrument cost analysis (pounds/annum)**

| Instrument | Vickers M–300 | Technicon SMAC | Hycel M | Technicon SMA II | | Technicon AA II | | LKB 8600 |
|---|---|---|---|---|---|---|---|---|
| Instrument associated costs | 50,736.60 | 50,955.05 | 45,671.46 | see below | | see below (c) | | (d) 2,150.00 |
| Type of workload | Profile | Profile | Total (a) | Profile | (b) Non batched | Batched | Non batched | Batched |
| Analyte | Analyte associated costs (pounds) | | | | | | | |
| Acid phosphatase | | | 9.43 | 553.89 | | 82.55 | | 111.01 |
| Albumin | 125.89 | 7.06 | 13.96 | 50.14 | | 2,202.20 | | |
| Alkaline phosphatase | 80.17 | 707.39 | 2,202.89 | 7,087.53 | | 6,171.95 | | 4,140.70 |
| Blank | 69.86 | | | | | | | |
| Amylase | | | 0.28 | | | | | |
| Aspartate transaminase | 9,550.39 | 256.52 | 999.96 | 3,849.37 | | 4,069.55 | | 3,910.48 |
| Blank | 9,550.39 | | | | | | | |
| Bicarbonate | 5,894.44 | 0.44 | 449.58 | 4.21 | 8.30 | 588.59 | 207.95 | |
| Blank | 96.05 | | | | | | | |
| Bilirubin (total) | 240.10 | 9.63 | 15.10 | 58.58 | 115.47 | 1,408.35 | 27.29 | |
| Bilirubin (conjugated) | 240.10 | 9.63 | 0.86 | 58.58 | 115.47 | 234.19 | 23.20 | |
| Blank | 240.10 | 9.63 | 15.10 | 58.58 | 115.47 | 1.408.35 | 27.29 | |
| Calcium | 169.30 | 14.26 | 13,45 | 43.08 | 84.92 | 1,593.72 | 100.15 | |
| Chloride | 38.80 | 1.30 | 0.25 | 9.93 | | 275.66 | | |
| Cholesterol | 594.22 | 93.34 | 1,242.01 | 133.69 | | 747.05 | | 2,012.84 |
| Creatine kinase | | 2,242.23 | 1,955.85 | 10,148.80 | | 3,193.13 | | 4,625.94 |
| Creatine | 58.98 | 1.65 | 2.32 | 4.37 | 8.62 | 744.33 | 3.43 | |
| Blank | 2.65 | | 0.10 | | | | | |
| Gamma glutamyl transferase | 15,695.66 | | 203.18 | | | | | 836.16 |
| Blank | 15,695.66 | | | | | | | |
| Glucose | 410.78 | 33.64 | 41.33 | 118.10 | 232.81 | 1.267.25 | 188.15 | |
| Iron | | | 4.54 | 45.78 | | 590.83 | | |
| Iron binding capacity | | | 4.54 | | | 590.83 | | |
| Lactate dehydrogenase | 12,588.72 | 735.53 | 52.84 | 6,655.89 | | 543.88 | | 324.01 |
| Blank | 81.87 | | | 44.59 | | 157.10 | | |
| Phosphate | 181.91 | 1.59 | 13.41 | 47.53 | | 1,525.63 | | |
| Potassium ) | 4.06 | 89.03 | 1.12 | 1.27 | 2.51 | 2,727.78 | 307.51 | |
| Sodium ) | | 71.03 | | | | | | |
| Total protein | 83.48 | 4.68 | 9.24 | 53.49 | | 2,218.37 | | |
| Blank | | 3.85 | | 43.71 | | 2,207.17 | | |
| Triglyceride | | 3,880.82 | 660.17 | 14,688.45 | | 2,049.05 | | 1,036.93 |
| Blank | | 2,994.51 | | | | | | |
| Urate | 93.33 | 77.81 | 4.34 | 316.02 | | 858.03 | | |
| Blank | 436.07 | | 20.30 | | | | | |
| Urea | 342.55 | 11.98 | 92.53 | 37.67 | 74.26 | 2,680.07 | 295.62 | |

to deal with the problem of cost changes due to inflation. Meanwhile it can be said that from the beginning of the last quarter of 1978 to the end of the first quarter of 1980, manpower costs have been increased by 25.2% based on the change in the mean salary of the MLSO and other costs by 24.5% based on the change in the retail price index.

ACKNOWLEDGEMENTS
The author wishes to thank Dr M. G. Rinsler and Dr F. L. Mitchell for helpful suggestions and discussion, the members of the Department of Clinical Chemistry at Northwick Park Hospital for their co-operation during data acquisition, and many colleagues in other laboratories and at the Department of Health and Social Security. He is indebted to representatives of the many instrument manufacturers involved in the study.

REFERENCES
[1] Coopers and Lybrand. Procedure for determining test costs in pathology laboratories, 1975, Department of Health and Social Security.
[2] DHSS Pathology Statistics Annual Report Form (SBH6).
[3] Clarke, A. A. and Dill, C. M., Final report on the SMAC costs in the Biochemistry Department of the Royal Victoria Hospital, Belfast, 1976, Department of Health and Social Security.
[4] Robinson, R. and Rock, P. Evaluation of DuPont automatic clinical analyser at George Eliot Hospital, Nuneaton, 1979, Department of Health and Social Security.
[5] Clark, A., Jones, P., Davies, A., Saunders, R. and Noon, D. A cost evaluation exercise of the centrifichem parallel fast analyser, 1977, Welsh Office.

### Key to Table 7.

(a) The Hycel–M is designed to be maintained in a standby mode and therefore batched and non-batched analyses may be costed together.
(b) The instrument associated costs for SMAII are £16,767.79 excluding pump tubes (£64.20 per channel) and capital depreciation and maintenance. These costs are dependent upon the number of channels and whether a flame unit is included. Figures are quoted below for 6 to 16 channels including flame unit.

| | |
|---|---|
| 6 channels | £9,990.00 |
| 7 channels | £10,836.00 |
| 8 channels | £11,470.00 |
| 9 channels | £12,633.00 |
| 10 channels | £14,324.00 |
| 11 channels | £15,487.00 |
| 12 channels | £16,544.00 |
| 13 channels | £17,390.00 |
| 14 channels | £18,553.00 |
| 15 channels | £19,187.00 |
| 16 channels | £19,610.00 |

The instrument associated costs cover profile and non-batched work as it is assumed that an instrument used for non-batched analysis would also be used for profiling. If the non-batched analysis is to be included however, there is an additional £1,546.00 to cover services, commercial sera and disposables costs.
(c) Instrument associated costs have been calculated for flame units and dual channel systems only, these are £2,118.89 and £1,637.35 per channel respectively.
(d) The cost of an LKB 2082–018 Kinetic Data Processor has been included under the capital.
(e) At the time this data was collated no information was available on the manufacturers maintenance contract. This has been assumed to be 5% of capital cost. Also, an allowance has been made in the capital cost to cover a micro-processor and interfacing equipment which were not currently available.
(f) This costing is based on the "fee per test" method and therefore there is no instrument associated cost.

| Coulter KEM–O–MAT | | Abbott VP | | Centrifichem 400 | | Du Pont ACA | |
|---|---|---|---|---|---|---|---|
| (e) 2,730.00 | | 6,649.43 | | 7,928.57 | | None (f) | |
| Batched | Non Batched | Batched | Non Batched | Batched | Non Batched | Batched | Non Batched |
| | | | | | | 897.92 | |
| 4.509.18 | | 2,319.67 | | 2,382.62 | | 27,308.25 | |
| 3,638.32 | | 2,144.10 | | 1,860.81 | | 18,247.29 | |
| | | 127.08 | 16.27 | | | 1,886.15 | 359.40 |
| 3,526.65 | | 2,116.90 | | 1,800.99 | | 18,409.77 | |
| | | 551.40 | 336.03 | | | 5,663.61 | 2,939.63 |
| 2,685.02 | 21.12 | 1,381.51 | 25.25 | 1,419.04 | 26.48 | 16,323.05 | 554.27 |
| 29.18 | 17.93 | 14.90 | 21.45 | 13.70 | 22.08 | 658.79 | 471.12 |
| 2,685.02 | 21.12 | | | 1,250.87 | 26.30 | | |
| 3,127.38 | 81.31 | 1,608.55 | 97.14 | 1,457.10 | 100.06 | 19,079.17 | 1,487.80 |
| 146.80 | | 80.78 | | | | 1,366.35 | |
| 1,158.67 | | 894.55 | | 1,180.00 | | 7,307.13 | |
| 1,988.43 | | 1,186.19 | | 1,725.57 | | 5,996.32 | |
| 1,226.54 | 3.13 | 631.20 | 3.75 | 571.04 | 3.88 | 7,813.24 | 83.71 |
| | | 204.81 | | 188.70 | | 2,149.22 | |
| 2,316.16 | 127.49 | 1,268.82 | 149.11 | 1,314.07 | 152.75 | 14,146.85 | 2,040.67 |
| | | 469.70 | | 556.94 | | 5,602.93 | |
| | | 469.70 | | 556.94 | | 5,602.93 | |
| 75.45 | | 42.82 | | 38.09 | | 892.38 | |
| 2,966.13 | | 1,141.08 | | | | 18,120.03 | |
| 4,488.53 | | 2,473.25 | | 2,090.41 | | 27,230.29 | |
| 440.11 | | 225.40 | | 182.97 | | 2,046.66 | |
| 1,258.99 | | 743.38 | | 691.85 | | 7,989.21 | |
| 1,266.19 | | | | 696.25 | | | |
| 5,618.37 | 247.18 | 2,891.60 | 294.01 | 2,534.61 | 302.20 | 33,791.85 | 3,617.93 |

